# Meta-Analysis of Integrated Therapeutic Methods in Noninvasive Lower Back Pain Therapy (LBP): The Role of Interdisciplinary Functional Diagnostics

**DOI:** 10.1155/2020/3967414

**Published:** 2020-03-19

**Authors:** Aleksandra Bitenc-Jasiejko, Krzysztof Konior, Danuta Lietz-Kijak

**Affiliations:** ^1^Department of Propedeutics, Physical Diagnostics and Dental Physiotherapy, Pomeranian Medical University in Szczecin, Szczecin, Poland; ^2^Medical Center in Nowogard, Nowogard, Poland

## Abstract

*Introduction*. Lower back pain (LBP) is almost a problem of civilizations. Quite often, it is a consequence of many years of disturbed distribution of tension within the human body caused by local conditions (injuries, hernias, stenoses, spondylolisthesis, cancer, etc.), global factors (postural defects, structural integration disorders, lifestyle, type of activity, etc.), or systemic diseases (connective tissue, inflammation, tumours, abdominal aneurysm, and kidney diseases, including urolithiasis, endometriosis, and prostatitis). Therefore, LBP rehabilitation requires the use of integrated therapeutic methods, combining the competences of interdisciplinary teams, both in the process of diagnosis and treatment. *Aim of the Study*. Given the above, the authors of the article conducted meta-analysis of the literature in terms of integrated therapeutic methods, indicating the techniques focused on a holistic approach to the patient. The aim of the article is to provide the reader with comprehensive knowledge about treating LBP using noninterventional methods. *Material and Methods*. An extensive search for the materials was conducted online using PubMed, the Cochrane database, and Embase. The most common noninterventional methods have been described, as well as the most relevantly updated and previously referenced treatment of LBP. The authors also proposed noninvasive (measurable) diagnostic procedures for the functional assessment of the musculoskeletal system, including initial, systematic, and cross-sectional control. All figures and images have been prepared by the authors and are their property.

**Results:**

This review article goes beyond combining a detailed description of each procedure with full references, as well as a comprehensive discussion of this very complex and troublesome problem.

**Conclusions:**

Lower back pain is a serious health problem, and this review article will help educate physicians and physiotherapists dealing with LBP in the options of evidence-based treatment. Ultimately, the article introduces and postulates the need to systematize therapeutic procedures in LBP therapy, with a long-term perspective.

## 1. Introduction

Lower back pain of mechanical origin is caused by injuries and/or degenerative conditions of the vertebrae and intervertebral discs and by the faulty distribution of forces within the soft tissues. The most frequently diagnosed mechanical problems are disc herniation (including disc fractures and their dehydration), compression fatigue fractures, and acute traumatic injuries [1]. In its chronic form, LBP affects over 20% of the global population; 24–80% of patients have a relapse of pain in the first year [2–5]. Despite the fact that 31% of patients with LBP demonstrate an improvement within 6 months, the recurrence of moderate pain is reported in 33% of cases, and acute pain is observed in 15% of sufferers within 1-2 years [6–8]. Recent studies indicate that people with LBP experience pain and the related disability longer than patients suffering from pain in the course of other diseases [9–11]. Nonspecific back pain is increasingly affecting young people, and this can be an important determinant of LBP in adulthood [12–15]. The necessity of systematization of therapeutic procedures results from the fact that back pain causes motor disability, thereby significantly reducing (and even temporarily disabling) motor activity with the consequent absence from work, particularly in the countries with a highly developed market economy [1, 16–18]. It has also been found that LBP, caused, for example, by discopathy, recurs after surgery [19–21]. LBP is a problem of civilizations, with epidemiological data clearly indicating the growth of the population affected by the condition. Therefore, it is important to take systematic actions in two areas:

- Differentiation of the problem, which should include both initial diagnosis and regular monitoring
- Systematizing nonsurgical and nonpharmacological therapeutic procedures, with particular emphasis on the roles played by interdisciplinary teams

Given the different aetiologies of LBP and severe pain, interdisciplinary teams, including doctors (imaging diagnostics and pharmacotherapy), physiotherapists, specialists in psychosomatic diseases (therapy of anxiety syndromes accompanied by chronic pain), dieticians (lifestyle, maintenance of normal body weight, and elimination of visceral problems, including intolerance, intoxication, and the presence of parasites), should be involved in the therapeutic process of back pain syndrome [22–29]. Scientific and research literature demonstrates, however, that evidence for multidisciplinary rehabilitation is rare, and physical and behavioural procedures are limited [30]. The impact of biopsychosocial factors on LBP, compared to biomechanical influences, is an important developmental aspect in research and scientific work [31]. Biological treatment is also in an experimental phase [32, 33].

The results of scientific and research studies are consistent in the conclusion that nonsteroidal anti-inflammatory drugs combined with muscle relaxants are effective methods of treating LBP [34–37]. Most literature sources indicate that staying in the reclined position has no effect on acute pain [38–43]. It has been repeatedly noted that the following factors play a role in the prevention of acute pain with mild aetiology:

- Education of people with LBP in methods to avoid overloading
- Moderate physical activity, using decompression movements in a position without pain or a significantly reduced pain, relaxing and stabilizing structures
- Return to normal activity [34, 35, 37, 39]

However, given the fact that, in most cases (i.e., 70% of people with LBP) complaints are caused by the dysfunction of myofascial structures, the most important scientific research issue should be the systematization of manual therapy (MT) procedures. The more so (apart from trauma, fatigue fractures, postural defects, and systemic diseases) in faulty force migration, arising from functional structure shortcuts, increased tension, fascia densification and tensegration disorders, and so on, are the primary causes of discopathy and degenerative conditions of the spine [1, 3, 44, 45].

This approach forces MT specialists to plan therapeutic procedures in a systematic way, starting from actions aimed at eliminating acute pain and ending with structural integration, reduction of abnormal compression forces, and consequently stabilization. Given that the therapeutic procedure (especially in the case of chronic pain) can be extended and periodic exacerbations are possible, in addition to imaging examinations, regular objectified monitoring of the patient's condition is necessary, the more so because there is also a literature discrepancy as to the use of imaging examinations [46–49]. According to guidelines, imaging diagnostics is not recommended in the following cases:

- Pain lasting less than six weeks: no red flags, the patient with LBP not having neurological symptoms (pain or numbness of the lower limbs, sciatica, and so on), and osteomyelitis is not suspected

Red flags include trauma, sudden weight loss, cancer, the long-term use of steroid drugs, increasing neurological symptoms, urinary incontinence, age over 70, and osteoporosis. A prolonged treatment lasting over 12 months is also a red flag [3, 50, 51]. Given the huge role of conservative therapy in LBP, the procedures used in the treatment of red flags should take into account the role of extended medical history taken by manual therapy specialists, educators, physiotherapists, etc. [52].

However, an imaging examination is the most important and objective diagnostic method to differentiate the problem of back pain of the spinal origin. Magnetic resonance imaging (MRI) is applied to assess the origin of neurological and soft tissue pain, while X-ray (X-ray/RTG) is used to evaluate posttraumatic bone and joint changes as well as abnormalities in the structure of the bones and their arrangement. Unfortunately, due to invasiveness and economic aspects, imaging examinations cannot be used to monitor the therapy. They are also not applicable in functional therapy and re-education of movement patterns, motor activity, etc.

A primary therapeutic task in LBP is to educate the patient on pain management. In addition to education on rest and physical activity, neurophysiological education is very important. This is a cognitive-behavioural intervention to provide knowledge of neurophysiology and neuroscience of pain in order to change beliefs about illness, disability, and above all adaptation to pain [53–55]. The management should differentiate the origin of pain, both in terms of mechanical (motor) stimulus and the protective (nociceptive) mechanism [56–60]. A literature study indicates a significant interaction between treatment conditions and the intensity of LBP, and thus physical fitness. Educational activities should be conducted in a clear and concise manner, adjusted to the intelligence, education, and expectations of patients. Particular emphasis should be put on the ability of patients to act independently in case of exacerbations. The family also plays an important role in the treatment [61–63].

### 1.1. Purpose of Meta-Analysis

The aim of the study is to review the literature on integrated therapeutic techniques used to treat LBP as well as to indicate the methods of postural and functional diagnostics and to provide the reader with comprehensive knowledge of the treatment of LBP using noninterventional methods.

## 2. Methodology of Meta-Analysis

The literature reviewed in this study covers the period of 21 years (1998–2019). The extensive search for materials was conducted online using PubMed, the Cochrane database, and Embase. The results were evaluated and checked for correct qualification, indicating and confirming the importance of combining different therapeutic methods of LBP and the relevance of interdisciplinary teams to be included in the LBP diagnosis and rehabilitation. The analysis covered 175 articles selected from a group of 1363 publications. All figures and images have been prepared by the authors and are their property.

## 3. The Merits of the Issue

### 3.1. Manual Therapy in LBP

Various therapeutic methods are used in the management of patients with LBP. However, we should pay attention to the procedures treating the functionality of the musculoskeletal system in a holistic way. The therapy based on structural integration is called tensegration. A role of the deep fascia in the aetiology of LBP was already indicated in 1939 [64]; modern medicine seeks one of the main causes of nontraumatic back pain in this structure [65–69]. Excessive tension of the tissues creates a damaging stimulus that is distributed within the human body in a linear manner [69–71]. Hence, pain may appear in a place distant from the site where the pain stimulus originally occurred [68, 72–75]. This indicates that the restoration of tension in anatomical tapes is essential for the process of rehabilitation, pain therapy, and restoring structural balance. As a result, greater strength is generated, and loads are transferred with the consequent shock absorption and the ability to support the muscles stabilizing the spine, which are closely connected with a given fascia tape [68, 76].

*The concept of fascial tapes* allows the connections between the myofascial lines causing dysfunction of the body posture to be determined, thereby including them in therapeutic procedures. This has a huge impact on the effectiveness of the therapy. The holistic approach to the fascial system is based on the current scientific research on fascial anatomy. A review of literature in this area clearly reveals that few authors treat fascia as a three-dimensional system [76–78]. Nevertheless, it has been repeatedly pointed out that manual therapy based on the concept of tensegration (structural integration) is one of the most effective methods of balancing tensions within the motor organ in LBP therapy [79, 80].

Osteopathic techniques: osteopathic manipulative treatment (OMT), used in patients with LBP, are integrated therapeutic methods taking into account various manual techniques to improve the functionality of the musculoskeletal structures and the entire body (structural and organ integration). Holistic therapy is based on, among other things, manipulation (OMT), including spine manipulation, muscle energy techniques (MET), visceral techniques, and exercises [81, 82]. It has been shown that OMT has a clinically significant effect on reducing pain and improving the functionality of the spine, lower limbs, and pelvis, both in patients with acute and chronic nonspecific LBP (including pregnant and postpartum women) [38].

Muscle energy technique (MET): muscle energy techniques have several applications. They can be used to lengthen the shortened muscle, strengthen the weakened muscle, and reduce local swelling and passive congestion. The technique is usually applied as part of a therapeutic complex used in acute LBP [83–85]. It also brings good results in hypomobility of the sacroiliac joints which affects LBP patients [86, 87].

*High velocity low amplitude (HVLA)* is a technique of short-lever manipulation of the joints that acts subtly and precisely. The method is based on unblocking the joint by applying a low amplitude and high velocity force. Previous reports provided little significant evidence that spine manipulation was better than other treatments for chronic low back pain [88, 89]. However, recent reviews suggest that spine manipulation is an option of pain management, though its effectiveness may vary depending on the duration of symptoms and the method of treatment [90, 91]. In the following example, manipulative and osteopathic techniques (HVLA) were combined with the SNAG techniques according to Mulligan's concept. This was done to improve the glide of the joints with the muscle function, and this distinguishes Mulligan's concept from other manipulative methods.

*AKA-H (Arthrokinematic Approach-Hakata)* method of manual therapy, developed by Setsuo Hakata in 1979, is used to treat abnormalities in joint movement. Scientific literature indicates that the effectiveness of the AKA-H method has been proven, both in the case of acute low back pain (as S. Hakata did), joint contractures, neuromuscular retraining, as well as in the case of chronic pain. The AKA-H method primarily includes techniques of neuromobilization and joint mobilization, including two procedures: manual therapy and physical therapy [92–98].

*Mulligan's concept* is another method complementing the therapy of LBP. SNAG (sustained natural apophyseal glide) is one of the most important MWM techniques. It involves the use of a passive glide on the vertebra and active movement made by the patient. The glide occurs in the articular plane, and the movement is performed in loading [99]. The reports indicate that the addition of the SNAG technique to other rehabilitation methods improves the range of spinal mobility and reduces pain [100–102]. The literature also indicates that compared to incorrect mobilization, the SNAG technique did not bring better results in terms of mobility [99]. According to the authors, no comment can be made on the results of this study because the technique was performed in asymptomatic patients. Konstantinou et al. demonstrated that MWM therapy significantly increased the average range of spinal motion compared to patients with LBP receiving a placebo [103].

Therapy of patients with LBP often requires the use of neuromobilization which is largely overlooked in various MT methods.

*Neuromobilization* allows nerve shifting in relation to the structures surrounding the neuronal tissue to be restored. It brings back the possibilities of stretching and tightening of the nerves. The technique should be used as early as possible when there are no irreversible morphological changes; it should cover all tissues affected by the pathology [104, 105].

### 3.2. Other Methods Used in LBP Therapy

*Acupuncture* is a nonspecific therapy having a large spectrum of indications. It activates central brain pathways, thus inhibiting pain reactions [106]. Research and scientific literature studies indicate the effectiveness of acupuncture in improving the function and relieving pain in LBP patients, but the results vary individually [107]. A review of studies gives no conclusive evidence that acupuncture is more effective in the treatment of chronic pain than a placebo or sham acupuncture [108–111]. The evidence for acupuncture effectiveness in LBP also remains controversial [112]. On the one hand, unambiguous points (meridians) relating to LBP were determined, and the number of people taking advantage of acupuncture has increased significantly in recent years [113–115]. On the other hand, similar to assessing the effectiveness of manual therapy, there are controversies over the methodology of scientific works, unjustified comparison of the study population [116, 117].

*Kinesio Taping (KT)* is a relatively new method, supporting the treatment of musculoskeletal disorders, including LBP. KT is not only sensory but also a proprioreceptive interaction. An appropriate application, adjusted to the patient's needs, allows the regeneration of the places affected by the disease and improves microcirculation. Moreover, the method has a positive effect on the normalization of the fascial system tension and a reduction of pressure on pain receptors. This results in folding the skin surface and increasing the space between the skin and fascia [118, 119]. The method improves blood and lymph microcirculation and activates self-healing processes. The literature indicates that the use of KT reduces pain and instability of the spine [120–122].

### 3.3. Rehabilitation Exercises and Autotherapy

The scientific literature describes many exercise methods used to reduce and prevent LBP [123, 124]. They are applicable both in acute pain, in autotherapy, and in stabilization of the musculoskeletal system.

*The McKenzie method* is designed to reduce the intensity of acute and chronic pain of the lower back and belongs to treatment systems focused on maintaining proper posture and consists of the repetition of the same movements. The McKenzie protocol is one of the most common recognized physiotherapeutic concepts used in patients with LBP [125, 126]. However, this method proved to be less effective compared to local stabilization training of the lumbar spine [127].

*Yoga* is a therapeutic method that conceptually combines psychoemotional and structural balance. It is a collection of many positions that supplement the therapy (stabilization) in LBP patients with both acute and chronic pain in a very positive way [128–130]. It also has a positive effect on respiratory functions and consequently a direct impact on the stability of the spine [131, 132].

### 3.4. Stabilization of the Patient's Condition and Stabilization Exercises

In recent years, the stability of the trunk (spinal cord) has been defined as the ability to maintain a stable neutral position of the spine. The global muscles participate in movements of the torso, while local muscles play an important stabilizing role. Stabilization training in patients with LBP reduces pain and instability as well as preventing relapses [133–135]. Hides et al. described how cricket players suffering from LBP were subjected to stability training (multifidus, transverse abdominal and pelvic floor muscles) with the consequent decrease in pain [136]. According to the literature, an ultrasound observation detected no advantage of stabilization exercises over general exercises [137]. There is also an opposing conclusion that stabilization exercises are more effective than standard training [138]. However, we must remember that the therapy aimed at unblocking the joints by correcting the muscle tension is complex. Searching for the best exercises, focused on TrA (transversus abdomnis) and MF (multifidus), should be done under ultrasound guidance. This is due to the fact that the correct contraction is a key to individual and personalized stabilization training, where proper mobility of the joints is required. It is easy to monitor therapeutic progress in this regard by measuring the thickness of a given muscle in an ultrasound, as indicated in the literature [139–141]. In special cases, an ultrasound can be used to evaluate the progress of therapy.

Even though the costs of magnetic resonance imaging, surgery, and corticosteroids have increased significantly, no improvement has been observed in the incidence of LBP and the consequent disability [48, 142]. Each LBP assessment procedure should begin with the taking of a detailed medical history and performing a physical examination, including identification of red flags. An MRI is one of the most useful diagnostic tools to evaluate the condition of the intervertebral discs that have a significant effect on kinematic patterns [143].

The physical examinations are used mainly to assess red flags, the quality of pain, and neurological symptoms and to perform visual evaluation of the quality of movement [144]. Given the relationship between functional disorders and nonspecific back pain, the authors believe in the importance of diagnostic procedures to evaluate the quality of movement and in the need to conduct scientific and research works in this area. Particularly, in the era of time-space research technologies, procedures should be focused on the implementation of measurable, objective, noninvasive, and economic rehabilitation methods [145–147].

### 3.5. Evidence for the Considerations above

**Figure 1:**
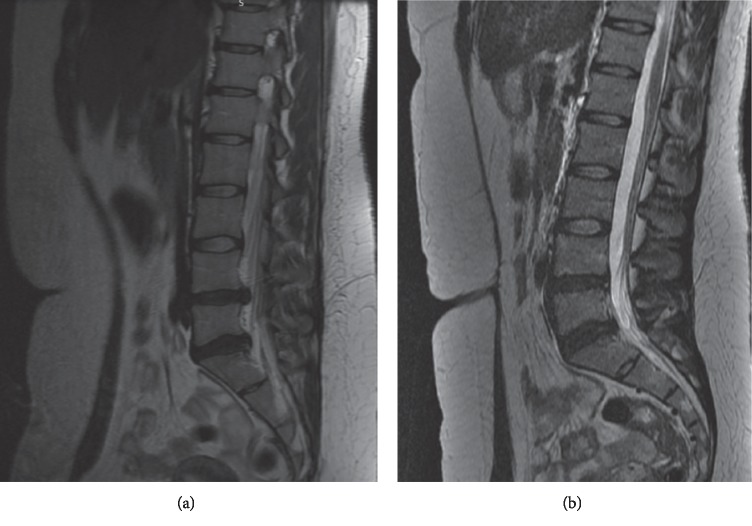
MRI. Date of examination: (a) 4^th^ December 2018 and (b) 20^th^ September 2019.

A reference to postural and functional diagnostics and integrated therapeutic procedures on the example of a 28-year-old patient who has suffered from pain since the age of 15. The medical history revealed that such an intensive acute LBP did not appear until the last two weeks. A magnetic resonance imaging visualized class IV changes in the intervertebral discs (Figures 1(a) and 1(b)) (according to the classification by Pfirrmann et al.) [148] in L3/L4 (protrusion) and L4/L5 (extrusion) (Figures 1(a) and 1(b)). After therapy lasting more than 8 months both results should be qualified as a protrusion, without migration into the spinal canal or pressure on the meningeal sac. According to the classification, the modic changes can be defined as type I, which is an important positive prognostic factor justifying the use of noninvasive therapeutic methods. However, at the same time, scientific literature indicates that, in the old age, there is a high risk of transformation of a type-I modic change into a type-II modic change [149, 150]. The incidence of LBP in modal changes ranges from 18% to 62% [151, 152]. Type II modic changes are the most common in segments L4/L5 and L5/S1, and disk degeneration is an important risk factor [125, 153, 154]. The condition of the disc is influenced by the supply of nutrients to the cartilaginous endplate that may be disturbed by biomechanical abnormalities in the lumbar region [153].

Given that the condition of the intervertebral discs is also influenced by spine kinematics, in addition to the evaluation of the disc itself, postural parameters and functionality of the musculoskeletal system are important diagnostic aspects. The initial evaluation and systematic monitoring of the patient were supplemented with computer diagnostic methods using the BIOMECH Studio program applied at each therapeutic visit. This aspect is relevant to the functional assessment, making examination results objective and measurable. The technique is also used to monitor the progress of rehabilitation procedures.

*The first stage of the therapeutic process* using manual therapy (MT) was pain relief. For this purpose, the techniques were applied to restore tension in individual anatomical chains (according to the concept of Anatomy Trains). The aim of the therapy was to integrate tensions within the myofascial structures in order to restore body balance and, as a consequence, corrective and stabilizing forces. At the same time, respiratory education and isolation of tonic deep muscle contraction were carried out with the patient lying on the side (this position was chosen for the examination because lying on the back caused pain). In the first stage of the therapy, the use of osteopathic craniosacral therapy was impossible due to pain in the position suitable for manipulation and intense pain-related anxiety. Instead, functional mobilization of structures was performed according to Mulligan, which gradually eliminated pain and increased the range of motion in the lumbar region (the examinations performed in painless positions or until pain occurred). Manual therapy also included neuromobilization. After each visit, the patient had breathing training (autotherapy) carried out at home and at work. Kinesio Taping was also applied after the therapy (the type of application was chosen depending on the patient's needs). This stage lasted three months, and there were exacerbations of pain caused by the lack of the patient's compliance to perform exercises regularly and due to the intensification of professional activities.

*The second stage of the therapy* was carried out in the absence of pain or/and its full control. The stage was focused on increasing mobility in the sacroiliac joints and facet joints of the spine. The HVLA osteopathic technique was used to precisely operate on the short lever and, consequently, avoid the need to involve other joints. At the same time, MET procedures were implemented, activating the work of the hip rotators. It was aimed at stabilizing the sacroiliac joints and increasing the range of motion in the hip joints. Mulligan mobilization was carried out throughout the entire stages I and II. The patient mastered deep muscle coordination with maintained respiration while standing. Autotherapy was enriched with education of the patient on the exercises using limbs. Their goal was to integrate the superficial muscles with cocontraction of the deep muscles. Kinesio Taping was also applied in order to maintain the effects of therapy. This stage lasted three months.

*The third stage of the therapy* consisted of postural and dynamic muscle training. The examination of the posture revealed numerous disorders, including pelvic drooping on one side, anterior tilt of the pelvis, functional knee and foot valgity, and first degree of longitudinal flat feet and transverse flat feet. Stabilization exercises have been enriched with training on unstable ground.

After this stage, the patient performed exercises aimed at learning the correct (ergonomic) gait pattern and relief positions (decompression). She also received orthopaedic insoles designed especially for her needs, to eliminate structural changes in the feet and knees and helping to maintain the correct gait pattern. The personalized orthopaedic insoles supported subsequent phases of gait, taking into account the patient's mobility; dynamic elements allowed for shock absorption in each phase of the gait.

The patient attended therapeutic visits for 8 months, with a frequency of once a week and a duration of 45 minutes. Diagnostic procedures were implemented at every stage of the therapy (at each visit). They included photogrammetric and videogrammetric methods implemented during the pedobarographic assessment performed while standing and walking (duration of static analysis was 20 seconds, and gait examination lasted for about 1–1.5 minutes). The examination of the range of motion and pelvic kinematics evaluation lasted approximately 3–5 min. The functionality examinations proposed by the authors are noninvasive and economical (inexpensive).

**Figure 2:**
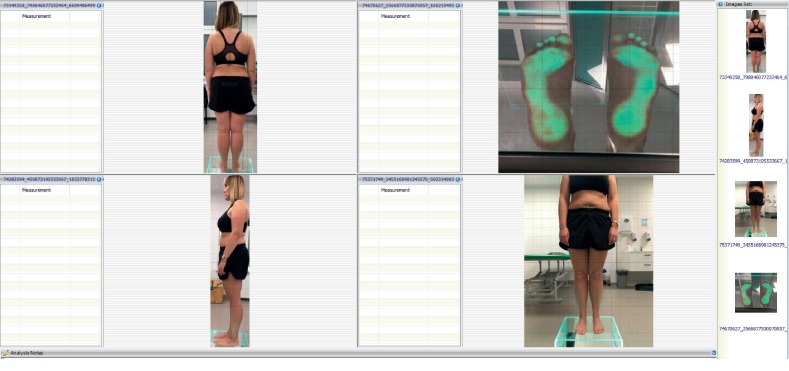
A photogrammetric examination performed using the BIOMECH Studio software.

*Procedure I*: photogrammetry and videogrammetry for anthropometry and physical examination (Figure 2).

**Figure 3:**
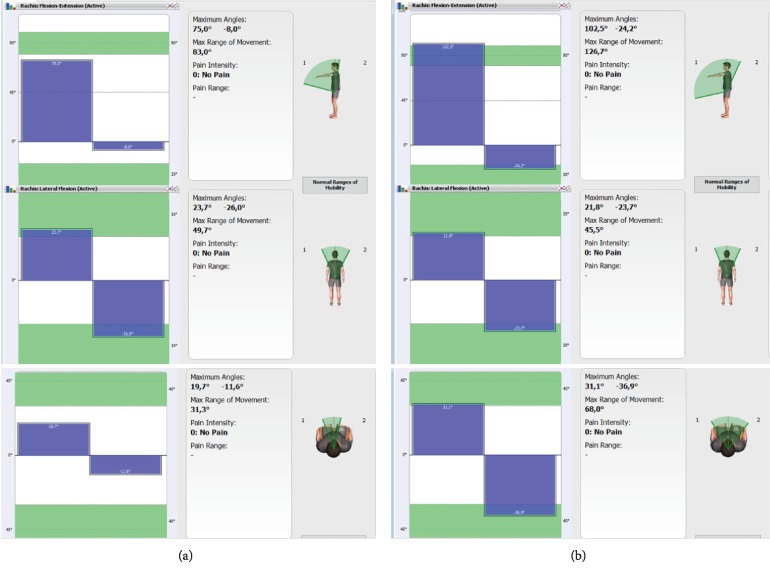
The exemplary result of the range of motion in the lumbar part, flexion/extension, lateral flexion to the left/right, and rotation to the left/right: the result (a) before a therapeutic session and (b) after a therapeutic session.

*Procedure II*: the examination of the range of motion in the joints using the WIVA Science® sensory-motor sensor. The examination was used to evaluate the functionality of structures as well as to regularly monitor the effectiveness of therapeutic methods. A user-friendly interface of the BIOMECH Studio software is also an important psychoemotional aspect for the patient, as the progress of the therapy can be observed on the TV screen while performing exercises (Figure 3).

Before and after each therapy, diagnostic procedures were implemented to assess the range of motion of the spine and hip joints. They allowed for the evaluation of the therapeutic process and effectiveness of the therapeutic method (i.e., maintaining the effects of therapy during a week break between each visit). An exemplary report is presented in Table 1.

**Figure 4:**
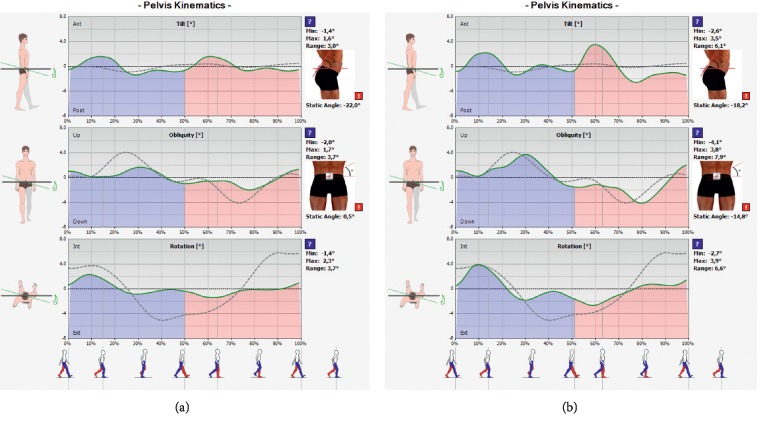
The exemplary result of pelvic parameters during standing and pelvic kinematics. The examination was performed at a therapeutic session: the result (a) before a therapeutic session and (b) after a therapeutic session.

The evaluation of pelvic kinematics and gait was also carried out using the Wiva Science® sensory-motor sensor. Pelvic mobility plays a pivotal role in maintaining balance of the neuromuscular system [155]. It is closely correlated with the quality of gait, mainly space-time parameters, which has been repeatedly demonstrated in physiological [156–158] and pathological gait [159–162]. The examination using the sensor allowed for the observation of the pelvis during walking and standing (including pelvic tilt angle) as well as for the analysis of pelvic movements in the three planes: anterior/posterior tilt, falling and lifting of the iliac alae, and rotation (Figures 4(a) and 4(b)).

The examination of the angle of lumbar lordosis under static and dynamic conditions is of great importance, mainly due to the close correlation between LBP and abnormalities of lumbar lordosis [163].

**Figure 5:**
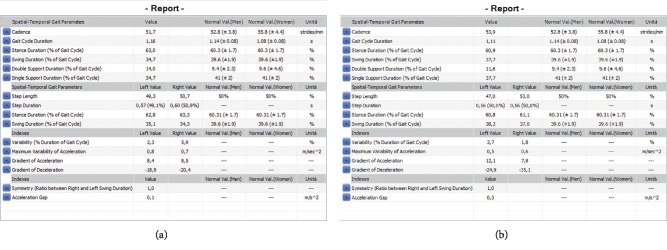
The exemplary result of a gait examination (a) before and (b) after a therapeutic session.

The exemplary results of gait analysis before and after therapy are shown in Figures 5(a) and 5(b).

**Figure 6:**
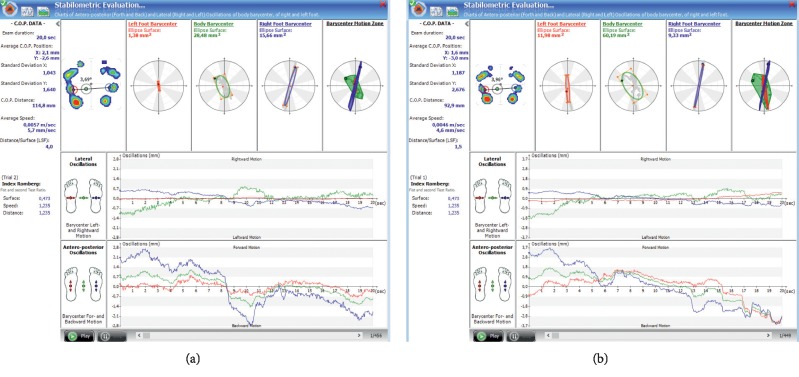
The exemplary result of a balance (stabilometric) examination performed at a therapeutic session.

The examination of postural functionality parameters can also be carried out using pedobarography, which allows for the static and dynamic examination from a perspective of the feet. The relationship between the function of the feet and LBP has been repeatedly indicated in the scientific and research literature [164]. A pedobarographic examination, which is a diagnostic method for the evaluation of balance (static test), is widely used in the assessment of the functional stability of the musculoskeletal system, particularly the body's ability to carry loads (static structural integration) and compensation (Figure 6) [165–169]. Stabilometry is an important aspect of functional diagnostics of the musculoskeletal system, and its inclusion in one device (i.e., in a pedobarograph) significantly reduces the cost of biomechanical diagnostics [170–177].

**Figure 7:**
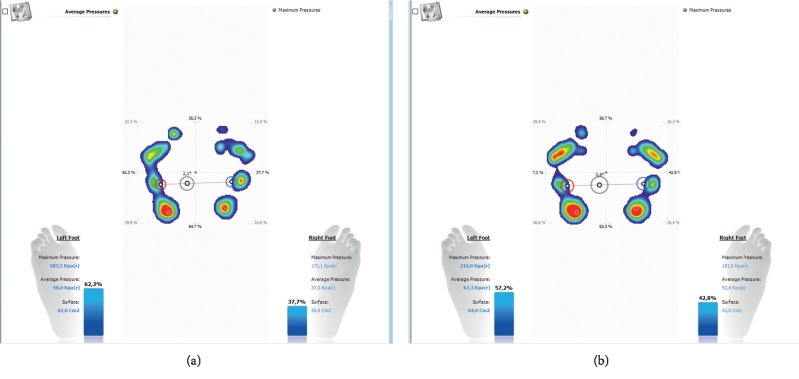
The exemplary result of the postural assessment of pressure distribution, in lateral and antero-posterior view measured (a) before and (b) after a therapeutic session.

Pedobarography also allows for the registration of the values of unit pressure, gait determinants, and distribution of pressure on the feet, making it applicable in the activities aimed at the early detection of postural threats [178]. Hence, the wide application of pedobarography includes orthopaedics, traumatology, rehabilitation of the musculoskeletal system, and in the assessment of functional disorders in spinal overload diseases. A pedobarographic examination also allows for the postural evaluation in the context of structural integration, i.e., testing the distribution of pressure on the right and left side and in an antero-posterior view. In patients with LBP, especially in the phase of pain, we observe compensatory disorders, causing the centre of gravity to be shifted to one limb, which in chronic conditions leads to postural overload disorders. The exemplary result of the general analysis of pressure distribution in the patient's body is presented in Figures 7(a) and 7(b) (the result obtained before and after a therapeutic session).

**Figure 8:**
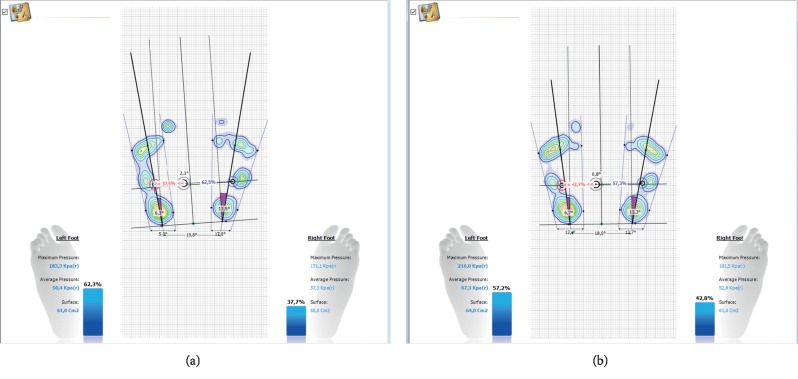
The exemplary result of assessing the mutual relationship between the feet and the proportions of the feet.

Pedobarography also allows for the assessment of the mutual relationship between the feet, angles of foot abduction, and proportions, which is presented in Figures 8(a) and 8(b). In the practice of the functional and postural assessment, the result should (and can) be correlated with the outcomes of hip joint mobility and the position of the lower extremities in the transverse plane. In line with the assumptions of structural integration, the result correlates with the position of the pelvis and lumbar lordosis.

**Figure 9:**
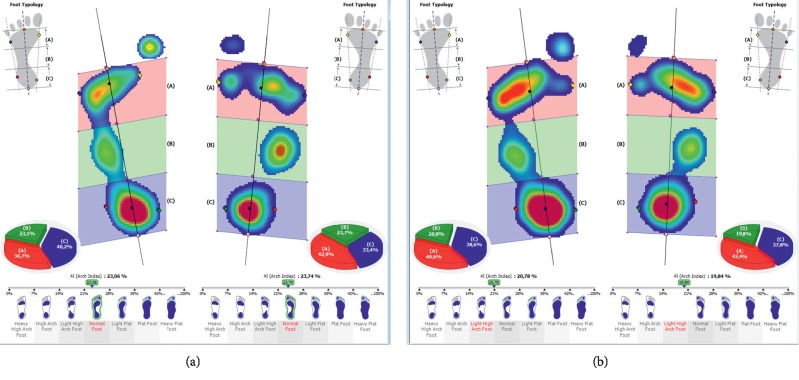
The exemplary result of the foot arch examination (a) before and (b) after a therapeutic session (the examination is performed while standing).

**Figure 10:**
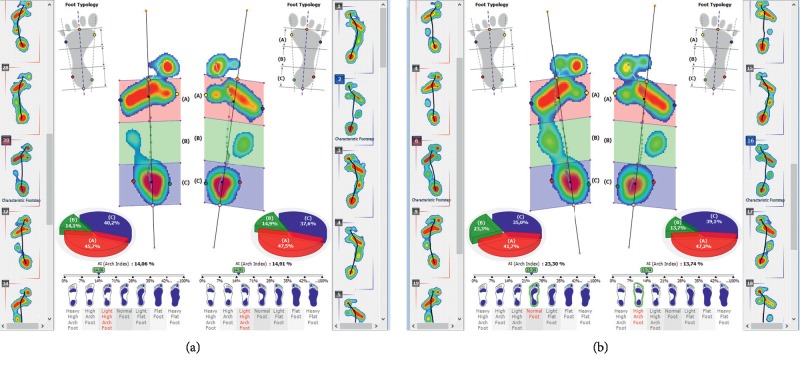
The exemplary result of the foot arch examination (AI index) (a) before and (b) after a therapeutic session (the examination is performed while walking).

A pedobarographic examination can also be used to evaluate the foot arch, both while standing (Figures 9(a) and 9(b)) and walking (Figures 10(a) and 10(b)).

**Figure 11:**
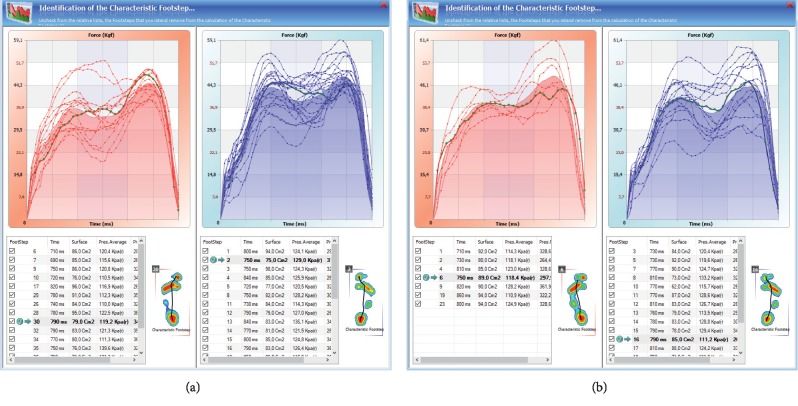
The exemplary graph presenting the results of strength distribution and time-space parameters during walking, measured at a therapeutic session.

The condition of the foot arch is of key importance to shock absorption during locomotion. The foot arch is the key to shock absorption during locomotion. A pedobarographic examination also allows for the observation of forces and time-space parameters during locomotion (Figures 11(a) and 11(b)).

**Figure 12:**
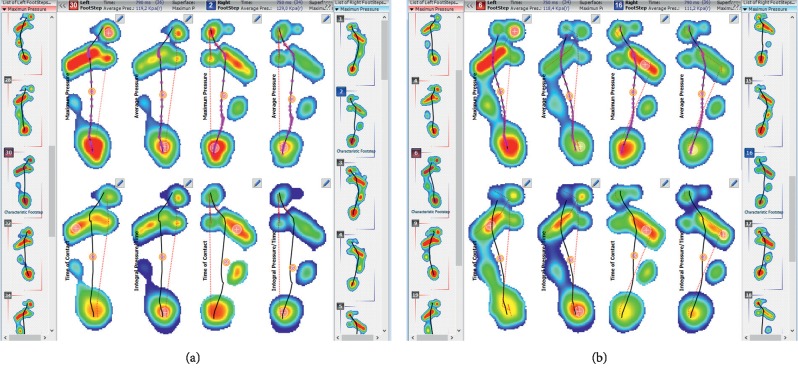
The exemplary result of the assessment of foot progression, carried out (a) before and (b) after a therapeutic session.

An important issue is also the function of changing the position from standing on the foot to standing on the toes (Figures 12(a) and 12(b)) which closely correlates with the mobility in the hips during walking and running, mainly due to supination and pronation of the foot.

## 4. Conclusions

1. The results of rehabilitation in LBP patients depend on comprehensively planned actions taken during treatment. The rehabilitation programme should include the knowledge of therapeutic methods used in specific functional disorders and give a chance to continue the exercises at home.
2. Given the variable course and diverse aetiology of LBP, therapeutic procedures should be based on holistic locomotor therapy.
3. The evaluation of therapy effectiveness ought to include a detailed functional analysis conducted at each stage of the treatment. Noninvasive computer diagnostic methods for biomechanical assessment of the musculoskeletal system should be recommended.
4. Vague results of the evaluation of the effectiveness of a given LBP manual therapy favour the use of a combination of methods as the application of one technique can eliminate just one of the restrictions.

## Figures and Tables

**Table 1:** The report generated before and after a therapeutic session.

Movement	Result before a therapeutic session	Result after a therapeutic session
Spinal flexion of the lumbar part	75.0	102.5
Spine extension of the lumbar part	8.0	24.2
Lateral bend of the spine to the left	23.7	21.8
Lateral bend of the spine to the right	26	23.7
Spinal rotation to the left in the lumbar part	19.7	31.1
Spinal rotation to the right in the lumbar part	11.6	36.9
Abduction in the left hip joint	27.8	41.5
Adduction in the left hip joint	26.7	26.5
Abduction in the right hip joint	27.9	35.2
Adduction in the right hip joint	24.5	25.3
Flexion in the left hip	79	96.9
Flexion in the right hip	81.3	103.6
Internal rotation in the left hip joint	25.7	25.1
External rotation in the left hip joint	46.9	59.5
Internal rotation in the right hip joint	27.4	39
External rotation in the right hip joint	45.8	54
Extension in the left hip joint	21.2	29.5
Extension in the right hip joint	15.4	29.5

## References

[1] Patrick N., Emanski E., Knaub M. A. (2014). Acute and chronic low back pain. *Medical Clinics of North America*.

[2] Balagué F., Mannion A. F., Pellisé F., Cedraschi C. (2012). Non-specific low back pain. *The Lancet*.

[3] Hoy D., Brooks P., Blyth F., Buchbinder R. (2010). The epidemiology of low back pain. *Best Practice & Research Clinical Rheumatology*.

[4] Henschke N., Maher C. G., Refshauge K. M. (2008). Prognosis in patients with recent onset low back pain in Australian primary care: inception cohort study. *BMJ*.

[5] Stanton T. R., Latimer J., Maher C. G., Hancock M. (2009). Definitions of recurrence of an episode of low back pain. *Spine*.

[6] Axén I., Leboeuf-Yde C. (2013). Trajectories of low back pain. *Best Practice & Research Clinical Rheumatology*.

[7] Stanton T. R., Henschke N., Maher C. G., Refshauge K. M., Latimer J., McAuley J. H. (2008). After an episode of acute low back pain, recurrence is unpredictable and not as common as previously thought. *Spine*.

[8] Carey T. S., Garrett J. M., Jackman A., Hadler N. (1999). Recurrence and care seeking after acute back pain. *Medical Care*.

[9] Vos T., Flaxman A. D., Naghavi M., Lozano R., Michaud C., Ezzat M. (2012). Years lived with disability (YLDs) for 1160 sequelae of 289 diseases and injuries 1990-2010: a systematic analysis for the Global Burden of Disease Study 2010. *Lancet*.

[10] Disease and Injury Incidence and Prevalence Collaborators (2016). Global, regional, and national incidence, prevalence, and years lived with disability for 310 diseases and injuries, 1990–2015: a systematic analysis for the Global Burden of Disease Study 2015. *Lancet*.

[11] Vos T., Flaxman A. D., Naghavi M. (2012). Years lived with disability (YLDs) for 1160 sequelae of 289 diseases and injuries 1990–2010: a systematic analysis for the Global Burden of Disease Study 2010. *The Lancet*.

[12] Pellisé F., Balagué F., Rajmil L. (2009 Jan). Prevalence of low back pain and its effect on health-related quality of life in adolescents. *Archives of Pediatrics & Adolescent Medicine*.

[13] Jeffries L. J., Milanese S. F., Grimmer-Somers K. A. (2007). Epidemiology of adolescent spinal pain. *Spine*.

[14] Coenen P., Smith A., Paananen M., O’Sullivan P., Beales D., Straker L. (2017). Trajectories of low back pain from adolescence to young adulthood. *Arthritis Care & Research*.

[15] Dionne C. E., Dunn K. M., Croft P. R. (2006). Does back pain prevalence really decrease with increasing age? A systematic review. *Age and Ageing*.

[16] Golob A. L., Wipf J. E. (2014). Low back pain. *Medical Clinics of North America*.

[17] Wipf S., Watkins R., Smith A., Schütze R., O’Sullivan P. (2013). Lives on hold. *The Clinical Journal of Pain*.

[18] Coggon D., Ntani G., Vargas-Prada S. (2013). International variation in absence from work attributed to musculoskeletal illness: findings from the CUPID study. *Occupational and Environmental Medicine*.

[19] Ohtori S., Koshi T., Yamashita M. (2011). Surgical versus nonsurgical treatment of selected patients with discogenic low back pain: a small-sized randomized trial. *Spine*.

[20] Hellum C., Johnsen L. G., Storheim K. (2011). Surgery with disc prosthesis versus rehabilitation in patients with low back pain and degenerative disc: two year follow-up of randomised study. *BMJ*.

[21] Jacobs W. C., van der Gaag N. A., Kruyt M. C. (2013). Total disc replacement for chronic discogenic low back pain: a cochrane review. *Spine*.

[22] Cherkin D. C., Sherman K. J., Balderson B. H. (2016). Effect of mindfulness-based stress reduction vs cognitive behavioral therapy or usual care on back pain and functional limitations in adults with chronic low back pain. *JAMA*.

[23] Kamper S. J., Apeldoorn A. T., Chiarotto A. (2014). Multidisciplinary biopsychosocial rehabilitation for chronic low back pain. *Cochrane Database of Systematic Reviews*.

[24] Klineberg E., Mazanec D., Orr D., Demicco R., Bell G., McLain R. (2007). Masquerade: medical causes of back pain. *Cleveland Clinic Journal of Medicine*.

[25] Jiménez-Sánchez S., Fernández-de-las-Peñas C., Carrasco-Garrido P. (2012). Prevalence of chronic head, neck and low back pain and associated factors in women residing in the autonomous region of Madrid (Spain). *Gaceta Sanitaria*.

[26] Linton S. J., Boersma K., Jansson M., Sv??rd L., Botvalde M. (2005). The effects of cognitive-behavioral and physical therapy preventive interventions on pain-related sick leave. *The Clinical Journal of Pain*.

[27] Camacho-Soto A., Sowa G. A., Perera S., Weiner D. K. (2012). Fear avoidance beliefs predict disability in older adults with chronic low back pain. *PM&R*.

[28] Basler H.-D., Luckmann J., Wolf U., Quint S. (2008). Fear-avoidance beliefs, physical activity, and disability in elderly individuals with chronic low back pain and healthy controls. *The Clinical Journal of Pain*.

[29] Lambeek L. C., van Mechelen W., Knol D. L., Loisel P., Anema J. R. (2010). Randomised controlled trial of integrated care to reduce disability from chronic low back pain in working and private life. *BMJ*.

[30] Zhao L., Manchikanti L., Kaye A. D., Abd-Elsayed A. (2019). Treatment of discogenic low back pain: current treatment strategies and future options-a literature review. *Current Pain and Headache Reports*.

[31] Cholewicki J., Breen A., Popovich J. M. (2019). Can biomechanics research lead to more effective treatment of low back pain? A point-counterpoint debate. *Journal of Orthopaedic & Sports Physical Therapy*.

[32] Peng B., Pang X. (2012). Regeneration and repair of intervertebral disc degeneration. *Regenerative Medicine in China*.

[33] Orozco L., Soler R., Morera C., Alberca M., Sánchez A., García-Sancho J. (2011). Intervertebral disc repair by autologous mesenchymal bone marrow cells: a pilot study. *Transplantation*.

[34] Hoiriis K. T., Pfleger B., McDuffie F. C. (2004). A randomized clinical trial comparing chiropractic adjustments to muscle relaxants for subacute low back pain. *Journal of Manipulative and Physiological Therapeutics*.

[35] Roelofs P. D., Deyo R. A., Koes B. W., Scholten R. J., van Tul-der M. W. (2008). Non-steroidal anti-inflammatory drugs for low back pain. *Cochrane Database of Systematic Reviews*.

[36] Hancock M. J., Maher C. G., Latimer J. (2007). Assessment of diclofenac or spinal manipulative therapy, or both, in addition to recommended first-line treatment for acute low back pain: a randomised controlled trial. *The Lancet*.

[37] Hancock M. J., Maher C. G., Latimer J., McLachlan A. J., Day R. O., Davies R. A. (2009). Can predictors of response to NSAIDs be identified in patients with acute low back pain?. *The Clinical Journal of Pain*.

[38] Franke H., Franke J. D., Fryer G. (2014). Osteopathic manipulative treatment for nonspecific low back pain: a systematic review and meta-analysis. *BMC Musculoskeletal Disorders*.

[39] Van Tulder M. W., Touray T., Furlan A. D., Solway S., Bouter L. M. (2003). Muscle relaxants for non-specific low back pain. *Cochrane Database of Systematic Reviews*.

[40] Dunsford A., Kumar S., Clarke S. (2011). Integrating evidence into practice: use of McKenzie-based treatment for mechanical low back pain. *Journal of Multidisciplinary Healthcare*.

[41] Machado G. C., Ferreira P. H., Yoo R. I. (2016). Surgical options for lumbar spinal stenosis. *The Cochrane Database of Systematic Reviews*.

[42] Delaney P. M., Hubka M. J. (1999). The diagnostic utility of McKenzie clinical assessment for lower back pain. *Journal of Manipulative and Physiological Therapeutics*.

[43] Hedlund R., Johansson C., Hägg O., Fritzell P., Tullberg T. (2016). The long-term outcome of lumbar fusion in the Swedish lumbar spine study. *The Spine Journal*.

[44] Casazza B. A. (2012). Diagnosis and treatment of acute low back pain. *American Family Physician*.

[45] Jarvik J. G., Deyo R. A. (2002). Diagnostic evaluation of low back pain with emphasis on imaging. *Annals of Internal Medicine*.

[46] Booth T. N., Iyer R. S., Falcone R. A. (2017). ACR appropriateness criteria back pain-child. *Journal of the American College of Radiology*.

[47] Henschke N., Maher C. G., Refshauge K. M. (2009). Prevalence of and screening for serious spinal pathology in patients presenting to primary care settings with acute low back pain. *Arthritis & Rheumatism*.

[48] Chou R., Fu R., Carrino J. A., Deyo R. A. (2009). Imaging strategies for low-back pain: systematic review and meta-analysis. *The Lancet*.

[49] Webster B. S., Cifuentes M. (2010). Relationship of early magnetic resonance imaging for work-related acute low back pain with disability and medical utilization outcomes. *Journal of Occupational and Environmental Medicine*.

[50] Downie A., Williams C. M., Henschke N. (2013). Red flags to screen for malignancy and fracture in patients with low back pain: systematic review. *BMJ*.

[51] Verhagen A. P., Downie A., Maher C. G., Koes B. W. (2017). Most red flags for malignancy in low back pain guidelines lack empirical support. *Pain*.

[52] Ferguson F. C., Morison S., Ryan C. G. (2015). Physiotherapists’ understanding of red flags for back pain. *Musculoskeletal Care*.

[53] Hodges P. W., Tucker K. (2011). Moving differently in pain: a new theory to explain the adaptation to pain. *Pain*.

[54] Taylor J. B., Goode A. P., George S. Z., Cook C. E. (2014). Incidence and risk factors for first-time incident low back pain: a systematic review and meta-analysis. *The Spine Journal*.

[55] Fillingim R. B. (2017). Individual differences in pain. *Pain*.

[56] O’Sullivan P., Waller R., Wright A. (2014). Sensory characteristics of chronic non-specific low back pain: a subgroup investigation. *Manual Therapy*.

[57] Hodges P. W., Smeets R. J. (2015). Interaction between pain, movement, and physical activity. *The Clinical Journal of Pain*.

[58] O’Sullivan P. (2005). Diagnosis and classification of chronic low back pain disorders: maladaptive movement and motor control impairments as underlying mechanism. *Manual Therapy*.

[59] Moseley G. L., Vlaeyen J. W. S. (2015). Beyond nociception. *Pain*.

[60] Rabey M., Beales D., Slater H., O’Sullivan P. (2015). Multidimensional pain profiles in four cases of chronic non-specific axial low back pain: an examination of the limitations of contemporary classification systems. *Manual Therapy*.

[61] Pires D., Cruz E. B., Caeiro C. (2015). Aquatic exercise and pain neurophysiology education versus aquatic exercise alone for patients with chronic low back pain: a randomized controlled trial. *Clinical Rehabilitation*.

[62] Nijs J., Paul van Wilgen C., Van Oosterwijck J., van Ittersum M., Meeus M. (2011). How to explain central sensitization to patients with “unexplained” chronic musculoskeletal pain: practice guidelines. *Manual Therapy*.

[63] Louw A., Diener I., Butler D. S., Puentedura E. J. (2011). The effect of neuroscience education on pain, disability, anxiety, and stress in chronic musculoskeletal pain. *Archives of Physical Medicine and Rehabilitation*.

[64] Kellgren J. H. (1939). On the distribution of pain arising from deep somatic structures with charts of segmental pain areas. *Clinical Science*.

[65] Schilder A., Hoheisel U., Magerl W., Benrath J., Klein T., Treede R.-D. (2014). Sensory findings after stimulation of the thoracolumbar fascia with hypertonic saline suggest its contribution to low back pain. *Pain*.

[66] Panjabi M. M. (2006). A hypothesis of chronic back pain: ligament subfailure injuries lead to muscle control dysfunction. *European Spine Journal*.

[67] Schleip R., Vleeming A., Lehmann-Horn F., Klingler W. (2007). Letter to the Editor concerning “A hypothesis of chronic back pain: ligament subfailure injuries lead to muscle control dysfunction” (M. Panjabi). *European Spine Journal*.

[68] Langevin H. M., Fox J. R., Koptiuch C. (2011). Reduced thoracolumbar fascia shear strain in human chronic low back pain. *BMC Musculoskeletal Disorders*.

[69] Masi A. T., Hannon J. C. (2008). Human resting muscle tone (HRMT): narrative introduction and modern concepts. *Journal of Bodywork and Movement Therapies*.

[70] Wilke J., Krause F., Vogt L., Banzer W. (2016). What is evidence-based about myofascial chains: a systematic review. *Archives of Physical Medicine and Rehabilitation*.

[71] Pardehshenas H., Maroufi N., Sanjari M. A., Parnianpour M., Levin S. M. (2014). Lumbopelvic muscle activation patterns in three stances under graded loading conditions: proposing a tensegrity model for load transfer through the sacroiliac joints. *Journal of Bodywork and Movement Therapies*.

[72] Kassolik K., Jaskólska A., Kisiel-Sajewicz K., Marusiak J., Kawczyński A., Jaskólski A. (2009). Tensegrity principle in massage demonstrated by electro- and mechanomyography. *Journal of Bodywork and Movement Therapies*.

[73] Kassolik K., Andrzejewski W. (2010). Tensegration massage. *Physiotherapy*.

[74] Piron A. (2007). The tensegrity concept applied to the laryngeal biodynamics. *Revue de Laryngologie–Otologie–Rhinologie*.

[75] Stecco C., Stern R., Porzionato A. (2011). Hyaluronan within fascia in the etiology of myofascial pain. *Surgical and Radiologic Anatomy*.

[76] Ajimsha M. S., Daniel B., Chithra S. (2014). Effectiveness of Myofascial release in the management of chronic low back pain in nursing professionals. *Journal of Bodywork and Movement Therapies*.

[77] Stecco A., Antonio S., Gilliar W. (2013). The anatomical and functional relation between gluteus maximus and fascia lata. *Journal of Bodywork and Movement Therapies*.

[78] Stecco C., Porzionato A., Macchi V. (2007). The expansions of the pectoral girdle muscles onto the brachial fascia: morphological aspects and spatial disposition. *Cells Tissues Organs*.

[79] Casato G., Stecco C., Busin R. (2019). Role of fasciae in nonspecific low back pain. *European Journal of Translational Myology*.

[80] Branchini M., Lopopolo F, Andreoli E., Loreti I., Marchand A. M., Stecco A. (2015). Fascial Manipulation® for chronic aspecific low back pain: a single blinded randomized controlled trial. *F1000Research*.

[81] Vaughan B., Morrison T., Buttigieg D., Macfarlane C., Fryer G. (2014). Approach to low back pain—osteopathy. *Australian Family Physician*.

[82] Franke H., Fryer G., Ostelo R. W. J. G., Kamper S. J. (2016). Muscle energy technique for non-specific low-back pain. A Cochrane systematic review. *International Journal of Osteopathic Medicine*.

[83] Wilson E., Payton O., Donegan-Shoaf L., Dec K. (2003). Muscle energy technique in patients with acute low back pain: a pilot clinical trial. *Journal of Orthopaedic & Sports Physical Therapy*.

[84] Patil P., Chandu B., Metgud S., Khatri S. (2010). Effectiveness of Muscle Energy Technique on quadratuslumborum in acute low back pain-randomized controlled trial. *Indian Journal of Physiotherapy and Occupational Therapy*.

[85] Selkow N., Grindstaff T., Cross K., Pugh K., Hertel J., Saliba S. (2009). Short-term effect of Muscle Energy Technique on pain in individuals with non-specific lumbopelvic pain: a pilot study. *The Journal of Manual & Manipulative Therapy*.

[86] Bindra S., Kumar M., Singh P., Singh J. (2012). A study on the efficacy of Muscle Energy Technique as compared to conventional therapy in chronic low back pain due to sacroiliac joint dysfunction. *Indian Journal of Physiotherapy and Occupational Therapy*.

[87] Dhinkaran M., Sareen A., Arora T. (2011). Comparative analysis of Muscle Energy Technique and conventional physiotherapy in treatment of sacroiliac joint dysfunction. *Indian Journal of Physiotherapy and Occupational Therapy*.

[88] Assendelft W. J. J., Morton S. C., Yu E. I., Suttorp M. J., Shekelle P. G. (2003). Spinal manipulative therapy for low back pain. *Annals of Internal Medicine*.

[89] Pasquier M., Daneau C., Marchand A.-A., Lardon A., Descarreaux M. (2019). Spinal manipulation frequency and dosage effects on clinical and physiological outcomes: a scoping review. *Chiropractic & Manual Therapies*.

[90] Furlan A. D., Yazdi F., Tsertsvadze A. (2012). A systematic review and meta-analysis of efficacy, cost-effectiveness, and safety of selected complementary and alternative medicine for neck and low-back pain. *Evidence-Based Complementary and Alternative Medicine*.

[91] Bronfort G., Haas M., Evans R. L., Bouter L. M. (2004). Efficacy of spinal manipulation and mobilization for low back pain and neck pain: a systematic review and best evidence synthesis. *The Spine Journal*.

[92] Kogure A., Kotani K., Katada S. (2015). A randomized, single-blind, placebo-controlled study on the efficacy of the arthrokinematic approach-hakata method in patients with chronic nonspecific low back pain. *PLoS One*.

[93] Williams P. L., Warwick R., Dyson M., Bannister L. H. (1989). *Gray’s Anatomy*.

[94] MacConaill M. A., Basmajian J. V. (1977). *Muscles and Movements: A Basis for Human Kinesiology*.

[95] Wyke B. (1981). The neurology of joints: a review of general principles. *Clinical Rheumatology*.

[96] Kaltenborn F. M. (1976). *Manual Therapy for the Extremity Joints, Specialized Techniques: Tests and Joint Mobilization*.

[97] Hakata S., Sumita K., Katada S. (2005). Wirksamkeit der AK-Hakata-Methode bei der Behandlung der akuten Lumbago. *Manuelle Medizin*.

[98] Rose S. J., Rothstein J. M. (1982). Muscle biology and physical therapy. *Physical Therapy*.

[99] Moutzouri M., Billis E., Strimpakos N., Kottika P., Oldham J. A. (2008). The effects of the Mulligan Sustained Natural Apophyseal Glide (SNAG) mobilisation in the lumbar flexion range of asymptomatic subjects as measured by the Zebris CMS20 3-D motion analysis system. *BMC Musculoskeletal Disorders*.

[100] Waqqar S., Shakil-ur-Rehman S., Ahmad S. (2016). McKenzie treatment versus mulligan sustained natural apophyseal glides for chronic mechanical low back pain. *Pakistan Journal of Medical Sciences*.

[101] Hussien H. M., Abdel-Raoof N. A., Kattabei O. M., Ahmed H. H. (2017). Effect of mulligan concept lumbar SNAG on chronic nonspecific low back pain. *Journal of Chiropractic Medicine*.

[102] May J., Krzyzanowicz R., Nasypany A., Baker R., Seegmiller J. (2015). Muligan concept use and clinical profile from the perspective of American certfied muligan practitioners mulligan concept use and clinical profile from the perspective of American certified mulligan practitioners. *Journal of Sport Rehabilitation*.

[103] Konstantinou K., Foster N., Rushton A., Baxter D., Wright C., Breen A. (2007). Flexion mobilizations with movement techniques: the immediate effects on range of movement and pain in subjects with low back pain. *Journal of Manipulative and Physiological Therapeutics*.

[104] Shacklock M. (2008). Neural mobilization: a systematic review of randomized controlled trials with an analysis of therapeutic efficacy. *Journal of Manual & Manipulative Therapy*.

[105] Scrimshaw S. V., Maher C. G. (2001). Randomized controlled trial of neural mobilization after spinal surgery. *Spine*.

[106] Oleson T. (2002). Auriculotherapy stimulation for neuro-rehabilitation. *NeuroRehabilitation*.

[107] Vickers A. J., Vertosick E. A., Lewith G. (2018). Acupuncture for chronic pain: update of an individual patient data meta-analysis. *The Journal of Pain*.

[108] Ezzo J., Berman B., Hadhazy V. A., Jadad A. R., Lao L., Singh B. B. (2000). Is acupuncture effective for the treatment of chronic pain? A systematic review. *Pain*.

[109] Yuan J., Purepong N., Kerr D. P., Park J., Bradbury I., Mcdonough S. (2008). Effectiveness of acupuncture for low back pain. *Spine*.

[110] Haake M., Müller H. H., Schadebrittinger C. (2007). German acupuncture trials (gerac) for chronic low back pain. *Archives of Internal Medicine*.

[111] Leibing E., Leonhardt U., Köster G. (2002). Acupuncture treatment of chronic low-back pain – a randomized, blinded, placebo-controlled trial with 9-month follow-up. *Pain*.

[112] Tulder Mv, Cherkin D. C., Berman B. (2000). Acupuncture for low back pain. *Cochrane Database of Systematic Reviews*.

[113] Lee I. S., Lee S. H., Kim S. Y., Lee H. J., Park H. J., Chae Y. Y. (2013). Visualization of the meridian system based on biomedical information about acupuncture treatment. *Evidence-Based Complementary and Alternative Medicine*.

[114] Shin J. Y., Ku B., Kim J. U. (2016). Short-term effect of laser acupuncture on lower back pain: a randomized, placebo-controlled, double-blind trial. *Evidence-Based Complementary and Alternative Medicine*.

[115] Lim T.-K., Ma Y., Berger F., Litscher G. (2018). Acupuncture and neural mechanism in the management of low back pain—an update. *Medicines*.

[116] Chia K. L. (2014). Electroacupuncture treatment of acute low back pain: unlikely to be a placebo response. *Acupuncture in Medicine*.

[117] Kim T. H., Kang J. W., Lee M. S. (2017). What is lost in the acupuncture trial when using a sham intervention?. *Acupuncture in Medicine*.

[118] Kase K., Wallis J., Kase T. (2003). *Clincal Thetapeutic Applications of the Kinesio Taping Method*.

[119] Neumann D. A. (2002). *Kinesiology of the Musculoskeletal System: Foundations for Physical Rehabilitation*.

[120] Abbasi S., Rojhani-Shirazi Z., Shokri E., García-Muro San José F. (2018). The effect of Kinesio Taping on postural control in subjects with non-specific chronic low back pain. *Journal of Bodywork and Movement Therapies*.

[121] Toprak Celenay S., Ozer Kaya D. (2019). Immediate effects of kinesio taping on pain and postural stability in patients with chronic low back pain. *Journal of Bodywork and Movement Therapies*.

[122] Bernardelli R. S., Scheeren E. M., Fuentes Filho A. R. (2019). Effects of Kinesio Taping on postural balance in patients with low back pain, a randomized controlled trial. *Journal of Bodywork and Movement Therapies*.

[123] Steffens D., Maher C. G., Pereira L. S. (2016). Prevention of low back pain: a systematic review and meta-analysis. *JAMA Internal Medicine*.

[124] Bernhardsson S., Oberg B., Johansson K., Nilsen P., Larsson M. E. (2015). Clinical practice in line with evidence? A survey among primary care physiotherapists in western Sweden. *Journal of Evaluation in Clinical Practice*.

[125] Cherkin D. C., Deyo R. A., Battla M. C., Street J. H., Hund M., Barlow W. (1998). A comparison of Physical therapy chiropractice manipulation or an educational booklet for the treatment of low back pain. *New England Journal of Medicine*.

[126] Machado L. A., de Souza M. S., Ferreira P. H., Ferreira M. L. (2006). The McKenzie method for low back pain: a systematic review of the literature with a meta-analysis approach. *Spine*.

[127] Hosseinifar M., Akbari M., Behtash H., Amiri M., Sarrafzadeh J. (2013). The effects of stabilization and mackenzie exercises on transverse abdominis and multifidus muscle thickness, pain, and disability: a randomized controlled trial in nonspecific chronic low back pain. *Journal of Physical Therapy Science*.

[128] Cramer H., Lauche R., Haller H., Dobos G. (2013). A systematic review and meta-analysis of yoga for low back pain. *The Clinical Journal of Pain*.

[129] Nambi G. S., Inbasekaran D., Khuman R., Devi S., Shanmugananth J. K. (2014). Changes in pain intensity and health related quality of life with Iyengar yoga in nonspecific chronic low back pain: a randomized controlled study. *International Journal of Yoga*.

[130] Bussing A., Ostermann T., Ludtke R., Michalsen A. (2012). Effects of yoga interventions on pain and pain‐associated disability: a meta‐analysis. *Journal of Pain*.

[131] Bahçecioğlu Turan G. (2019). The effect of yoga on respiratory functions, symptom control and life quality of asthma patients: a randomized controlled study. *Complementary Therapies in Clinical Practice*.

[132] Cramer H., Ward L., Steel A., Lauche R., Dobos G., Zhang Y. (2016). Prevalence, patterns, and predictors of yoga use: results of a U.S. nationally representative survey. *American Journal of Preventive Medicine*.

[133] Jackson J. K., Shepherd T. R., Kell R. T. (2011). The influence of periodized resistance training on recreationally active males with chronic nonspecific low back pain. *Journal of Strength and Conditioning Research*.

[134] Noormohammadpour P., Kordi M., Mansournia M., Akbari-Fakhrabadi M., Kordi R. (2018). Exercise program in the treatment of nurses with chronic low back pain: a single-blinded randomized controlled trial. *Asian Spine Journal*.

[135] Shamsi M., Sarrafzadeh J., Jamshidi A., Arjmand N., Arjmand F. (2017). Comparison of spinal stability following motor control and general exercises in nonspecific chronic low back pain patients. *Clinical Biomechanics*.

[136] Hides J. A., Stanton W. R., McMahon S., Sims K., Richardson C. (2008). Effect of stabilization training on multifidus muscle cross-sectional area among young elite cricketers with low back pain. *Journal of Orthopaedic & Sports Physical Therapy*.

[137] Shamsi M., Sarrafzadeh J., Jamshidi A., Zarabi V., Pourahmadi M. R. (2016). The effect of core stability and general exercise on abdominal muscle thickness in non-specific chronic low back pain using ultrasound imaging. *Physiotherapy Theory and Practice*.

[138] Waseem Akhtar M., Karimi H., Amir Gilani S. (2017). Effectiveness of core stabilization exercises and routine exercise therapy in management of pain in chronic non-specific low back pain: a randomized controlled clinical trial. *Pakistan Journal of Medical Sciences*.

[139] Wilson A., Hides J. A., Blizzard L. (2016). Measuring ultrasound images of abdominal and lumbar multifidus muscles in older adults: a reliability study. *Manual Therapy*.

[140] Wallwork T. L., Hides J. A., Stanton W. R. (2007). Intrarater and interrater reliability of assessment of lumbar multifidus muscle thickness using rehabilitative ultrasound imaging. *Journal of Orthopaedic & Sports Physical Therapy*.

[141] Stokes M., Rankin G., Newham D. J. (2005). Ultrasound imaging of lumbar multifidus muscle: normal reference ranges for measurements and practical guidance on the technique. *Manual Therapy*.

[142] Jarvik J. G., Hollingworth W., Martin B. (2003). Rapid magnetic resonance imaging vs radiographs for patients with low back pain: a randomized controlled trial. *JAMA*.

[143] Zou J. l, Yang H., Miyazaki M. (2009). Dynamic bulging of intervertebral discs in the degenerative lumbar spine. *Spine*.

[144] Suzuki H., Kanchiku T., Imajo Y., Yoshida Y., Nishida N., Taguchi T. (2016). Diagnosis and characters of non-specific low back pain in Japan: the yamaguchi low back pain study. *PLoS One*.

[145] Deng W., Papavasileiou I., Qiao Z., Zhang W., Lam K. Y., Han S. (2018). Advances in automation technologies for lower extremity neurorehabilitation: a review and future challenges. *IEEE Reviews in Biomedical Engineering*.

[146] Tavares C., Domingues M. F., Frizera-Neto A. (2018). Gait shear and plantar pressure monitoring: a non-invasive OFS based solution for e-health architectures. *Sensors*.

[147] Ferber R., Osis S. T., Hicks J. L., Delp S. L. (2016). Gait biomechanics in the era of data science. *Journal of Biomechanics*.

[148] Pfirrmann C. W. A., Metzdorf A., Zanetti M. (2001). Magnetic resonance classification of lumbar intervertebral disc degeneration. *Spine*.

[149] Braithwaite I., White J., Saifuddin J. (1998). Vertebral end-plate (Modic) changes on lumbar spine MRI: correlation with pain reproduction at lumbar discography. *European Spine Journal*.

[150] Kleinstuck E., Dvorak J., Mannion A. F. (2006). Are “structural abnormalities” on magnetic resonance imaging a contraindication to the successful conservative treatment of chronic nonspecific low back pain?. *Spine*.

[151] Allegri M., Montella S., Salici F. (2016). Mechanisms of low back pain: a guide for diagnosis and therapy. *F1000Research*.

[152] Rahme R., Moussa R., Bou-Nassif R. (2010). What happens to Modic changes following lumbar discectomy? Analysis of a cohort of 41 patients with a 3- to 5-year follow-up period. *Journal of Neurosurgery: Spine*.

[153] Chen Y., Bao J., Yan Q., Wu C., Yang H., Zou J. (2019). Distribution of Modic changes in patients with low back pain and its related factors. *European Journal of Medical Research*.

[154] Kuisma M., Karppinen J., Niinimaki J. (2006). A three years follow-up of lumbar spine endplate (Modic) changes. *Spine*.

[155] Lewis C. L., Laudicina N. M., Khuu A., Loverro K. L. (2017 Apr). The Human Pelvis: variation in structure and function during gait. *The Anatomical Record*.

[156] Bugané F., Benedetti M. G., Casadio G. (2012). Estimation of spatial-temporal gait parameters in level walking base on a single accelerometer: validation on normal subjects by standard gait analysis. *Comput Methods Programs Biomed*.

[157] Bonnet V., Mazzà C., McCamley J., Cappozzo A. (2013). Use of weighted Fourier linear combiner filters to estimate lower trunk 3D orientation from gyroscope sensors data. *Journal of NeuroEngineering and Rehabilitation*.

[158] Mazzà C., Donati M., McCamley J., Picerno P., Cappozzo A. (2012). An optimized Kalman filter for the estimation of trunk orientation from inertial sensors data during treadmill walking. *Gait & Posture*.

[159] Gaston M. S., Rutz E., Dreher T., Brunner R. (2011). Transverse plane rotation of the foot and transverse hip and pelvic kinematics in diplegic cerebral palsy. *Gait & Posture*.

[160] Goujon-Pillet H., Sapin E., Fodé P., Lavaste F. (2008). Three-dimensional motions of trunk and pelvis during transfemoral amputee gait. *Archives of Physical Medicine and Rehabilitation*.

[161] Tranberg R., Zugner R., Karrholm J. (2011). Improvements in hip- and pelvic motion for patients with osseointegrated trans-femoral prostheses. *Gait & Posture*.

[162] Rusaw D., Ramstrand N. (2011). Motion-analysis studies of transtibial prosthesis users: a systematic review. *Prosthetics and Orthotics International*.

[163] Chun S. W., Lim C. Y., Kim K., Hwang J., Chung S. G. (2017). The relationships between low back pain and lumbar lordosis: a systematic review and meta-analysis. *The Spine Journal*.

[164] O’Leary C. B., Cahill C. R., Robinson A. W., Barnes M. J., Hong J. (2013). A systematic review: the effects of podiatrical deviations on nonspecific chronic low back pain. *Journal of Back and Musculoskeletal Rehabilitation*.

[165] Akbari M., Karimi H., Farahini H., Faghihzadeh S. (2006). Balance problems after unilateral lateral ankle sprains. *Rehabilitation Research & Development*.

[166] Bennett P. J., Patterson C., Wearing S., Baglioni T. (1998). Development and Validation of a questionnaire designed to measure foot-health status. *American Podiatric Medical Association*.

[167] Błaszczyk J. W., Michalski A. (2006). Ageing and postural stability. *Studies in Physical Culture and Tourism*.

[168] Herrington L., Hatcher J., Hatcher A., McNicholas M. A. (2009). Comparison of Star Excursion Balance Test reach distances between ACL deficient patients and asymptomatic controls. *Knee*.

[169] Souchard P. (2011).

[170] Lawson T., Morrison A., Blaxland S., Wenman M., Schmidt C. G., Hunt M. A. (2015). Laboratory-based measurement of standing balance in individuals with knee osteoarthritis: a systematic review. *Clinical Biomechanics*.

[171] Geldhof E., Cardon G., De Bourdeaudhuij I. (2006). Static and dynamic standing balance: test-retest reliability and reference values in 9 to 10-year-old children. *European Journal of Pediatrics*.

[172] Skopljak A., Muftic M., Aziz S., Izet Masic, Zunic L. (2014). Pedobarography in diagnosis and clinical application. *Acta Informatica Medica*.

[173] Choi Y. R., Lee H. S., Kim D. E., Lee D. H., Kim J. M., Ahn J. Y. (2014). The diagnostic value of pedobarography. *Orthopedics*.

[174] Scoppa F., Capra R., Gallamini M., Shiffer R. (2013). Clinical stabilometry standardization. Basic definitions—acquisition interval—sampling frequency. *Gait & Posture*.

[175] Tamburella F., Scivoletto G., Iosa M., Molinari M. (2014). Reliability, validity, and effectiveness of center of pressure parameters in assessing stabilometric platform in subjects with incomplete spinal cord injury: a serial cross-sectional study. *Journal of NeuroEngineering and Rehabilitation*.

[176] Santos B. R., Delisle A., Larivière C., Plamondon A., Imbeau D. (2008). Reliability of centre of pressure summary measures of postural steadiness in healthy young adults. *Gait & Posture*.

[177] Duffell L. D., Gulati V., Southgate D. F. L., McGregor A. H. (2013). Measuring body weight distribution during sit-to-stand in patients with early knee osteoarthritis. *Gait & Posture*.

[178] Geil M. D., Lay A. (2004). Plantar foot pressure responses to changes during dynamic trans-tibial prosthetic alignment in a clinical setting. *Prosthetics and Orthodcs International*.

